# Supplementary material for: Neoadjuvant Treatment with HER2-Targeted Therapies in HER2-Positive Breast Cancer: A Systematic Review and Network Meta-Analysis
Source: Cancers (Basel). 2022 Jan 21;14(3):523. doi: 10.3390/cancers14030523 (PMC8833584; doi:10.3390/cancers14030523)
Supplement: Supplementary file 1 [file cancers-14-00523-s001.zip › cancers-1554233-supplementary.pdf]

Supplementary Materials

Neoadjuvant Treatment with HER2 Targeted Therapies in HER2 Positive Breast Cancer: A Systematic Review and Network Meta-Analysis

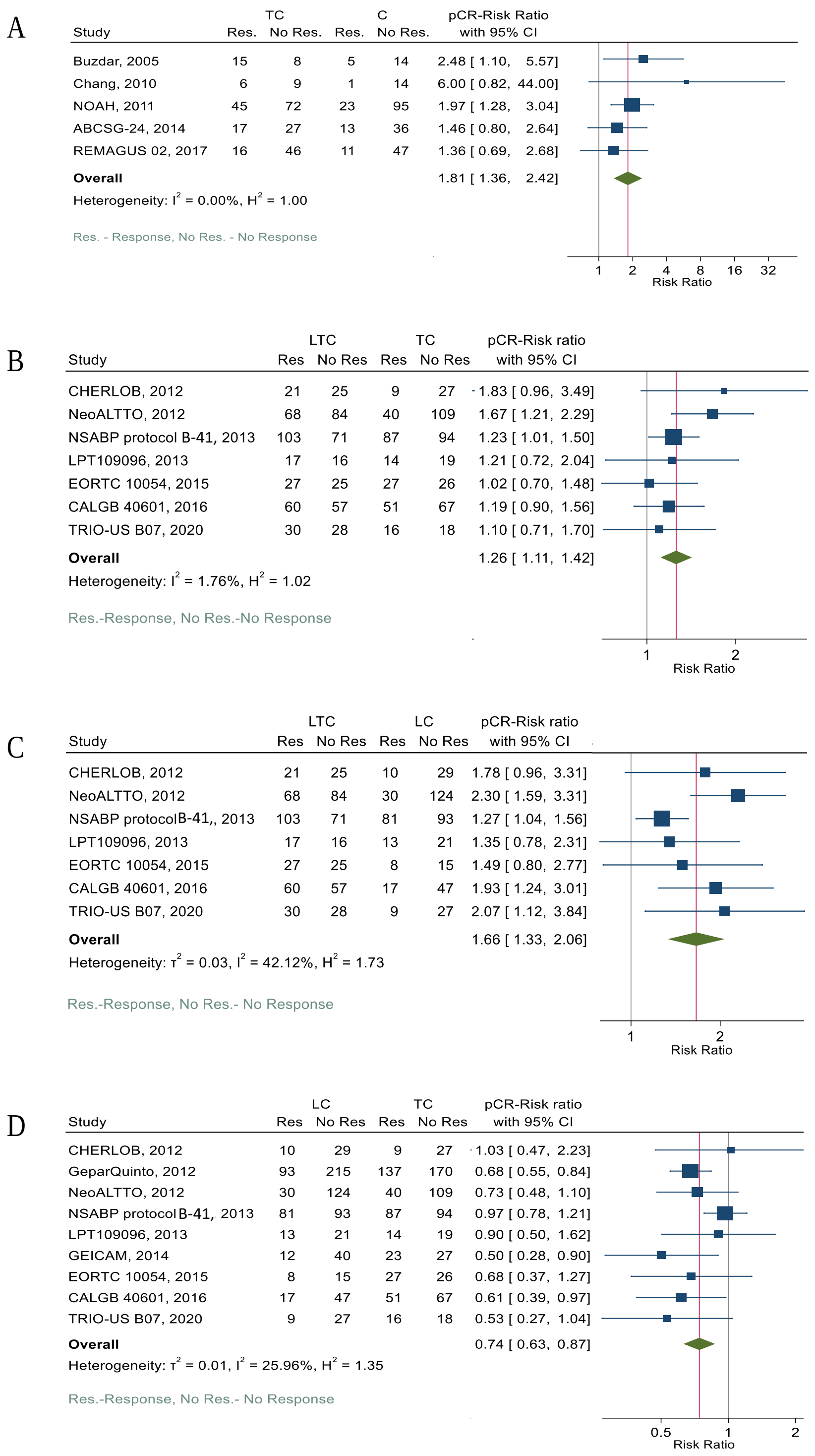

Figure S1. Forest plots of pairwise meta-analyses for pathological complete response (pCR). (**A**) TC vs C; (**B**) LTC vs TC; (**C**) LTC vs LC; (**D**) LC vs TC. C = Chemotherapy; TC = Trastuzumab + chemotherapy; LTC = Lapatinib + trastuzumab +chemotherapy; LC = Lapatinib + chemotherapy.

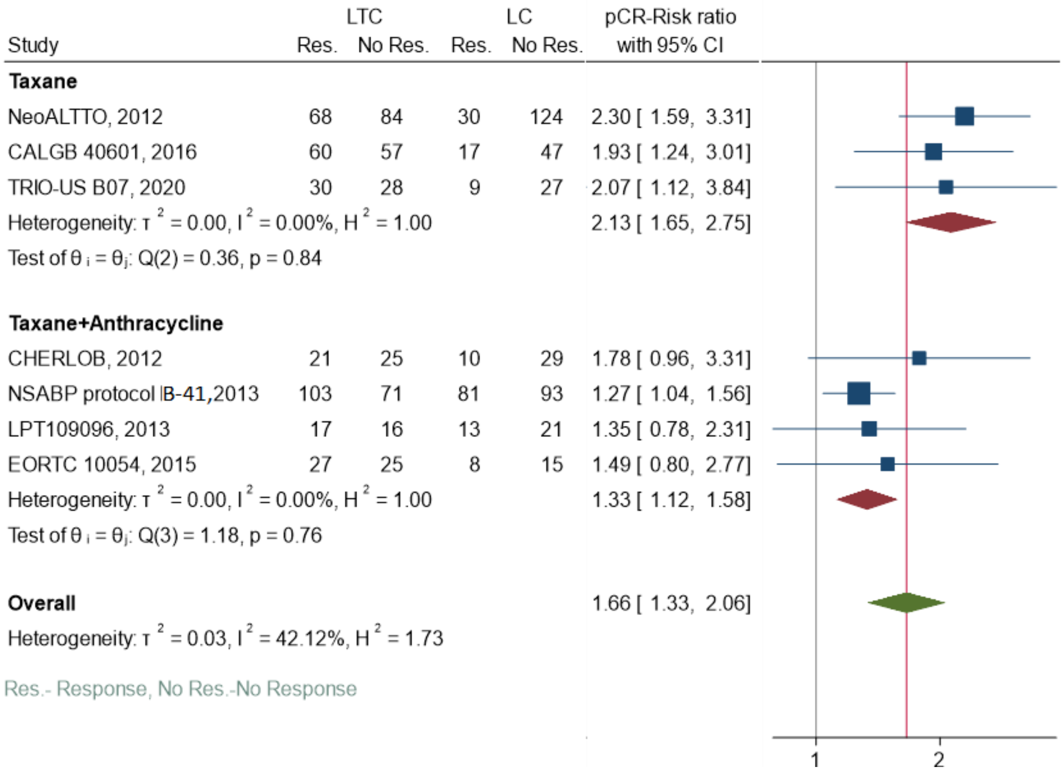

Figure S2. Subgroup analysis of pathological complete response (pCR) in LTC vs. LC according to types of chemotherapy. LTC = Lapatinib + trastuzumab +chemotherapy; LC = Lapatinib + chemotherapy.

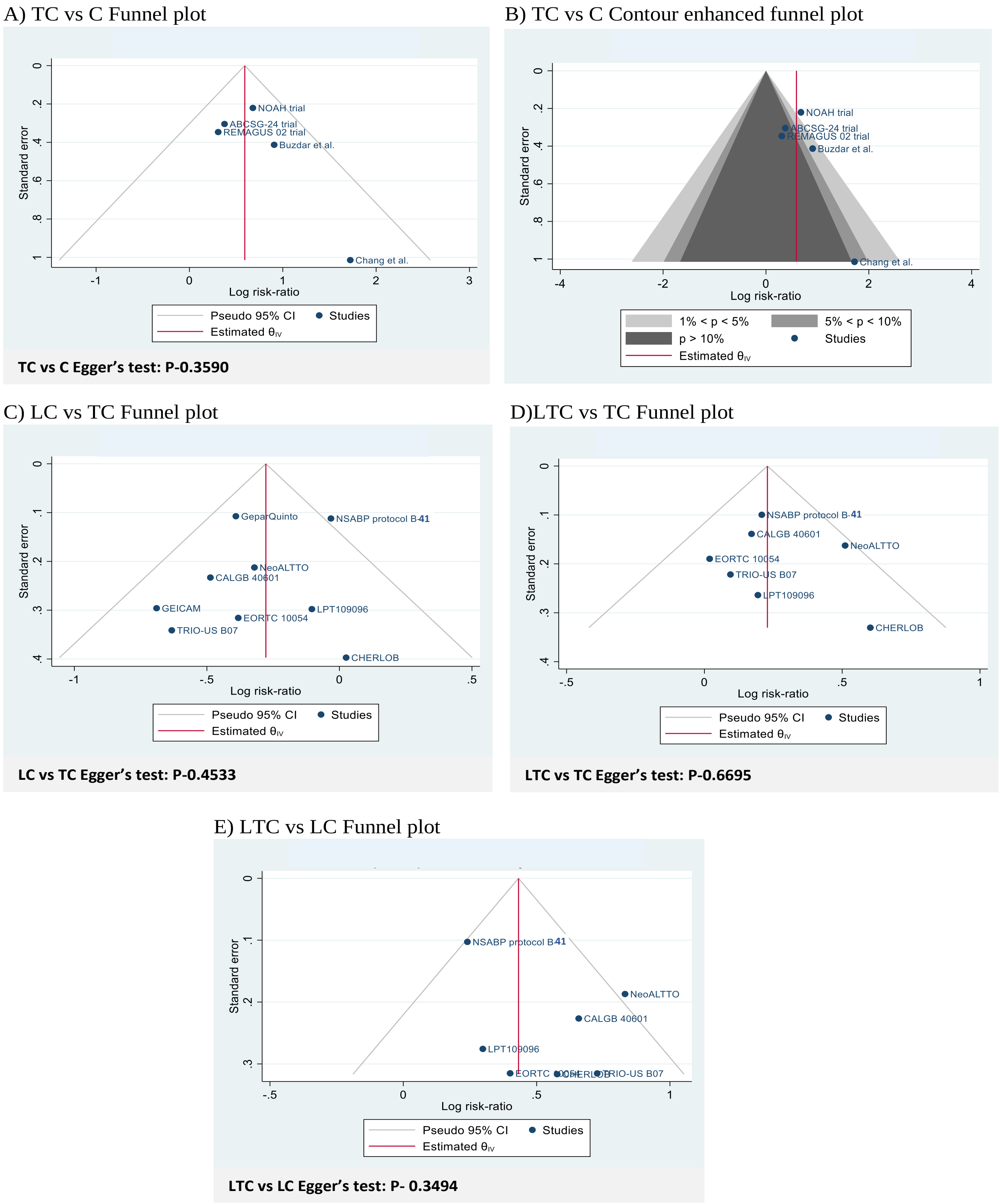

Figure S3. Funnel plots and contour enhanced funnel plots of pairwise meta-analyses comparisons for pathological complete response (pCR). (**A**) TC vs C Funnel plot; (**B**) TC vs C Contour enhanced funnel plot; (**C**) LC vs TC Funnel plot; (**D**) LTC vs TC Funnel plot; (**E**) LTC vs LC Funnel plot. C = Chemotherapy; TC = Trastuzumab + chemotherapy; LTC = Lapatinib + trastuzumab +chemotherapy; LC = Lapatinib + chemotherapy.

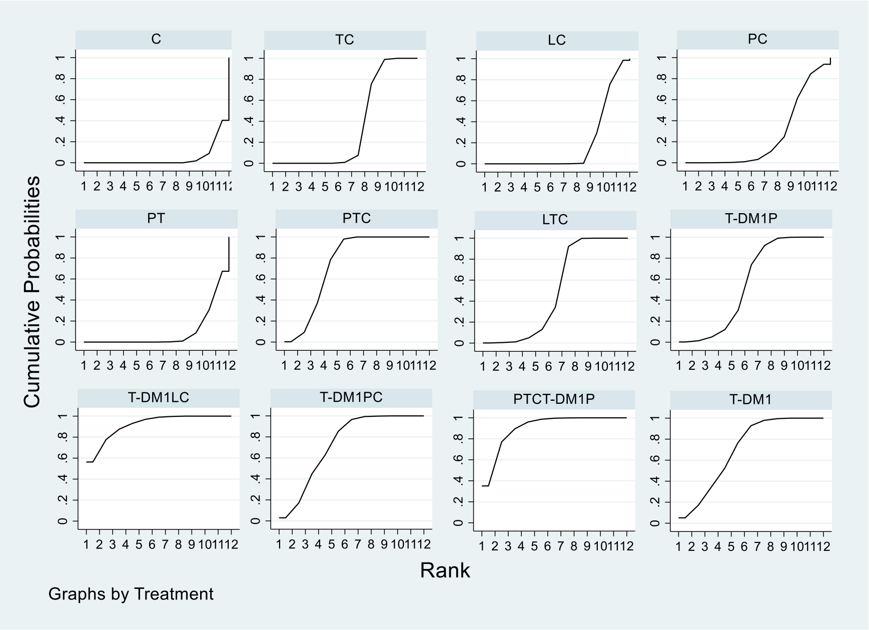

Treatment	SUCRA	PrBest	MeanRank
C	4.6	0	11.5
TC	34.8	0	8.2
PC	25.5	0	9.2
LC	18.6	0	10
PT	9.8	0	10.9
T-DM1P	55.8	0.2	5.9
PTC	74.8	0.3	3.8
LTC	49.7	0.1	6.5
T-DM1LC	91.9	56.3	1.9
T-DM1PC	73.5	2.9	3.9
PTC_T-DM1P	90.5	35.1	2
T-DM1	70.5	5.1	4.2

Figure S4. Surface under the cumulative ranking curves (SUCRA) for pathological complete response (pCR). C = Chemotherapy; TC = Trastuzumab + chemotherapy, PC = Pertuzumab + chemotherapy; LC = Lapatinib + chemotherapy; PTC = Pertuzumab + trastuzumab + chemotherapy; PT = Pertuzumab + trastuzumab; T-DM1LC = Trastuzumab emtansine + lapatnib + chemotherapy; T-DM1PC = Trastuzumab emtansine + pertuzumab + chemotherapy; PTC_T-DM1P = Pertuzumab + trastuzumab + chemotherapy followed by trastuzumab emtansine + pertuzumab; T-DM1 = Trastuzumab emtansine.

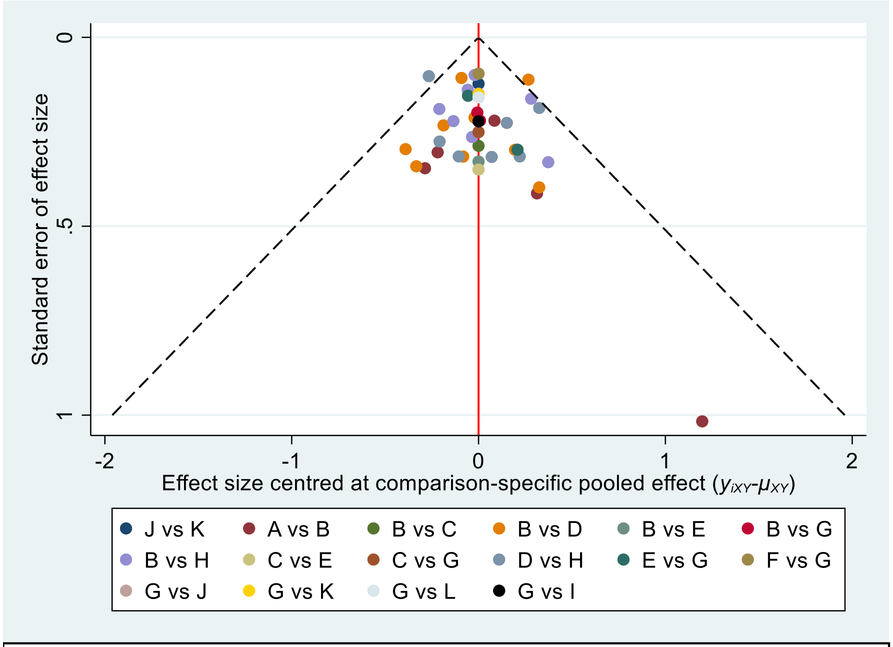

A: C (1), B: TC (2), C: PC (3), D: LC (4), E: PT (5), F: T-DM1P (6), G: PTC (7), H: LTC (8), I: T-DM1LC (9), J:T-DM1PC (10), K:PTC_T-DM1P (11), L:T-DM1 (12)

Figure S5. Comparison adjusted funnel plot of network meta-analysis of pathological complete response (pCR). C = Chemotherapy; TC = Trastuzumab + chemotherapy; LTC = Lapatinib + trastuzumab +chemotherapy; LC = Lapatinib + chemotherapy; T-DM1LC = trastuzumab emtansine + lapatnib + chemotherapy; T-DM1PC = Trastuzumab emtansine + pertuzumab + chemotherapy; T-DM1P = Trastuzumab emtansine + pertuzumab; PTC_T-DM1P = pertuzumab + trastuzumab + chemotherapy followed by trastuzumab emtansine + pertuzumab; T-DM1 = Trastuzumab emtansine.

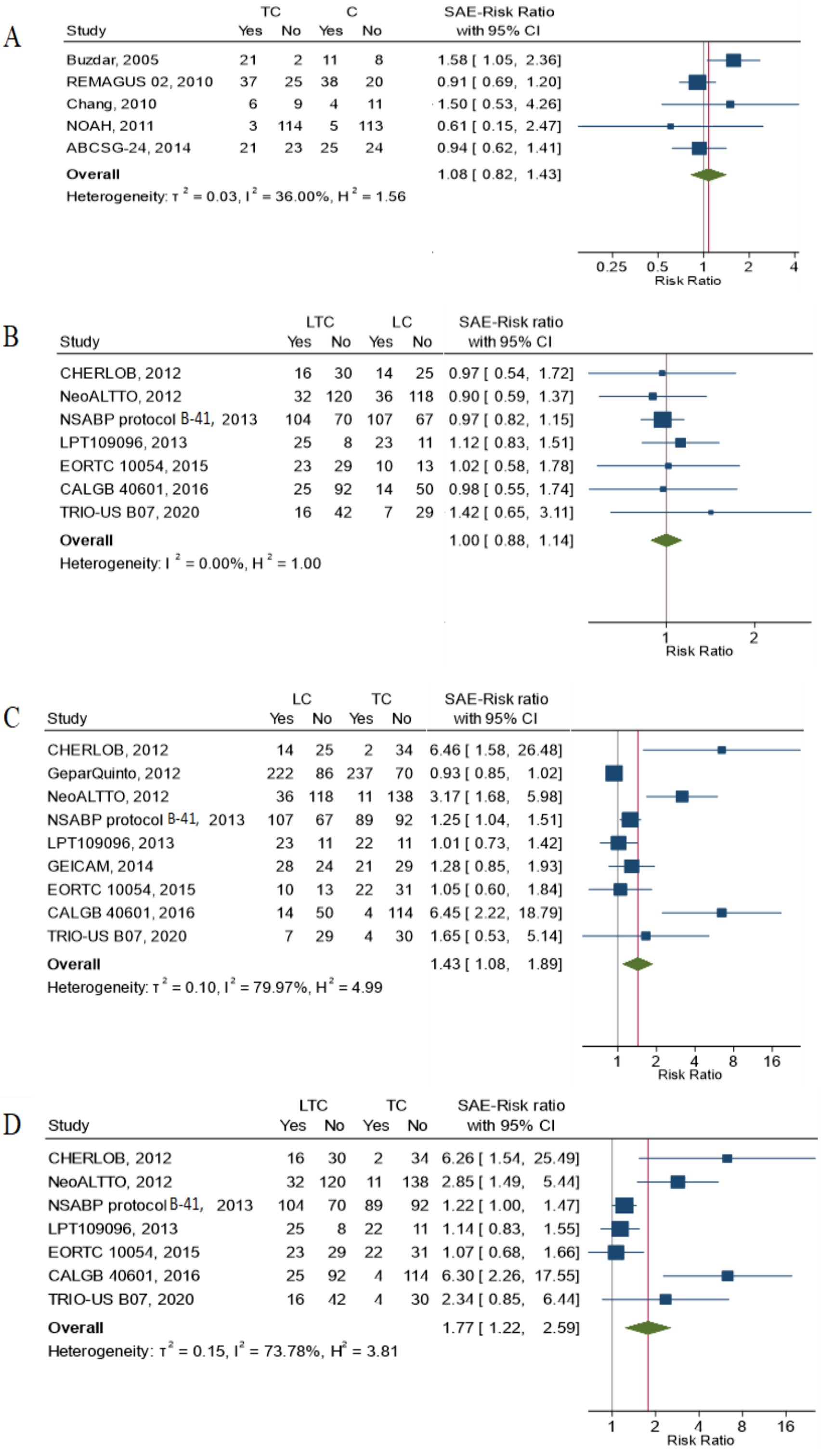

Figure S6. Forest plots of pairwise meta-analyses for serious adverse events (SAE). (**A**) TC vs C; (**B**) LTC vs LC; (**C**) LC vs TC; (**D**) LTC vs TC. C = Chemotherapy; TC = Trastuzumab + chemotherapy; LTC = Lapatinib + trastuzumab +chemotherapy; LC = Lapatinib + chemotherapy.

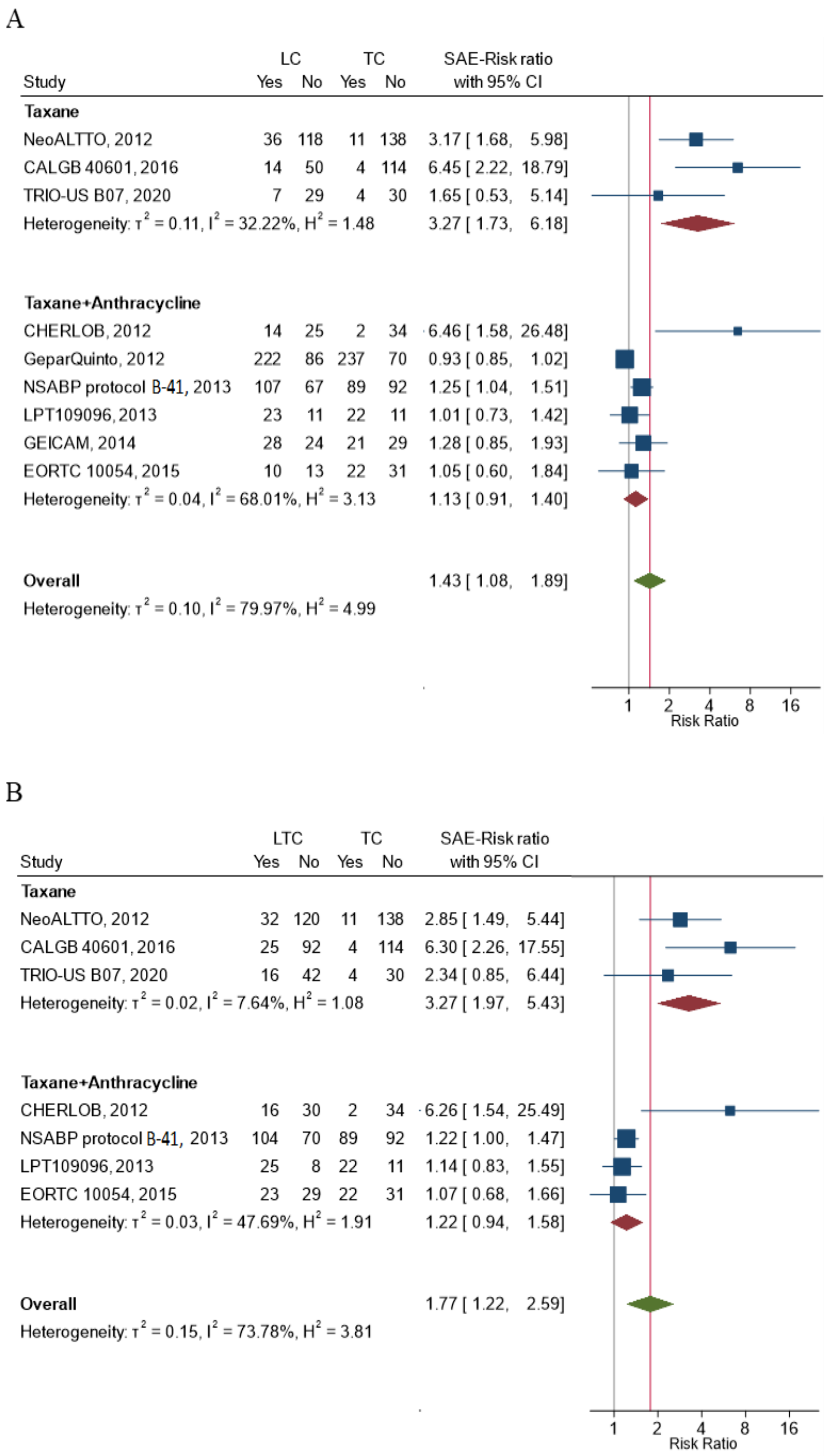

Figure S7. Subgroup analyses according to types of chemotherapy for serious adverse events (SAE). (**A**) LC vs TC; (**B**) LTC vs TC. C = Chemotherapy; TC = Trastuzumab + chemotherapy; LTC = Lapatinib + trastuzumab +chemotherapy; LC = Lapatinib + chemotherapy.

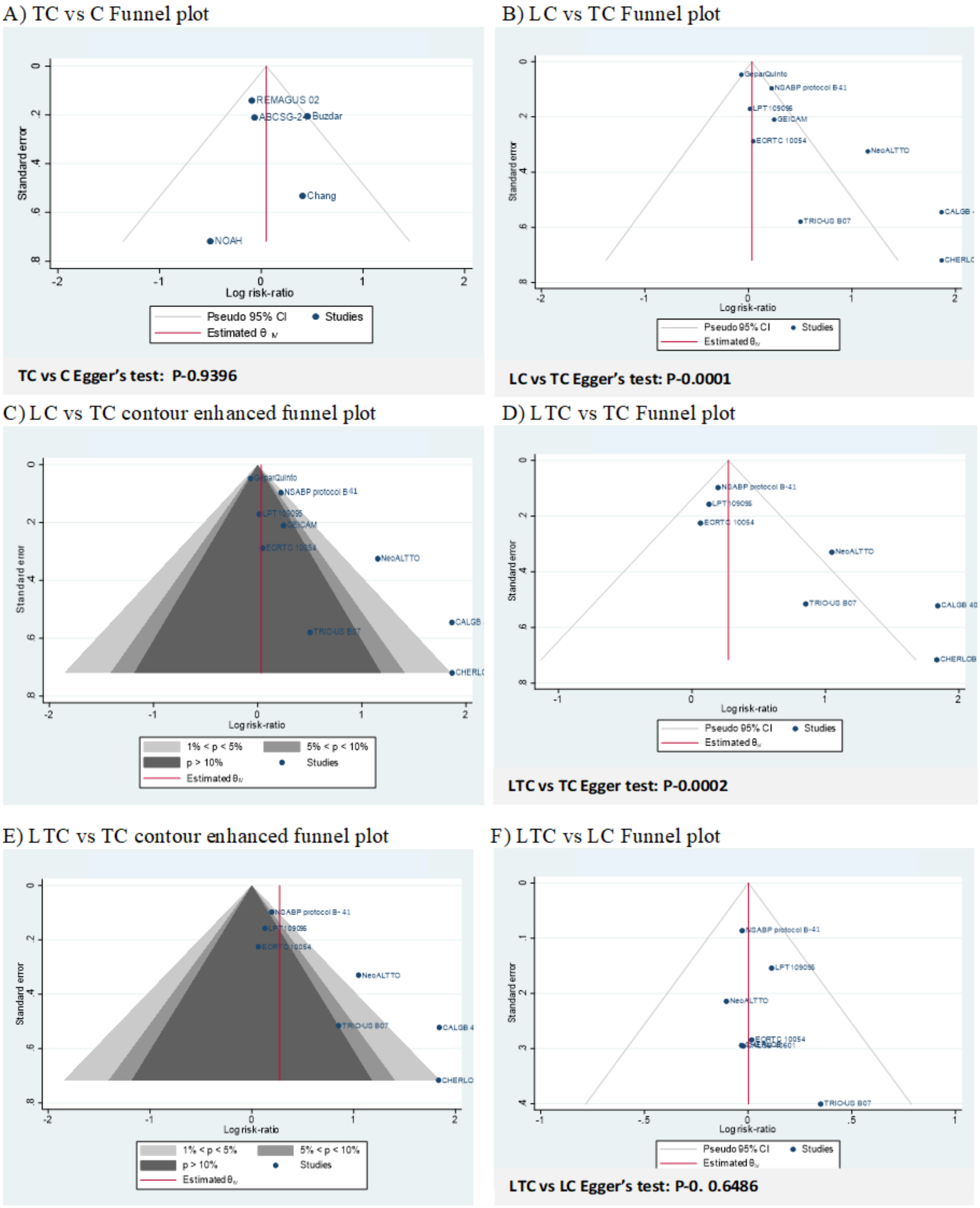

Figure S8. Funnel plots and contour enhanced funnel plots of pairwise meta-analyses for serious adverse events (SAE). (**A**) TC vs C Funnel plot; (**B**) LC vs TC Funnel plot; (**C**) LC vs TC Contour enhanced funnel plot; (**D**) LTC vs TC Funnel plot; (**E**) LTC vs TC Contour enhanced funnel plot; (**F**) LTC vs LC Funnel plot. C = Chemotherapy; TC = Trastuzumab + chemotherapy; LTC = Lapatinib + trastuzumab +chemotherapy; LC = Lapatinib + chemotherapy.

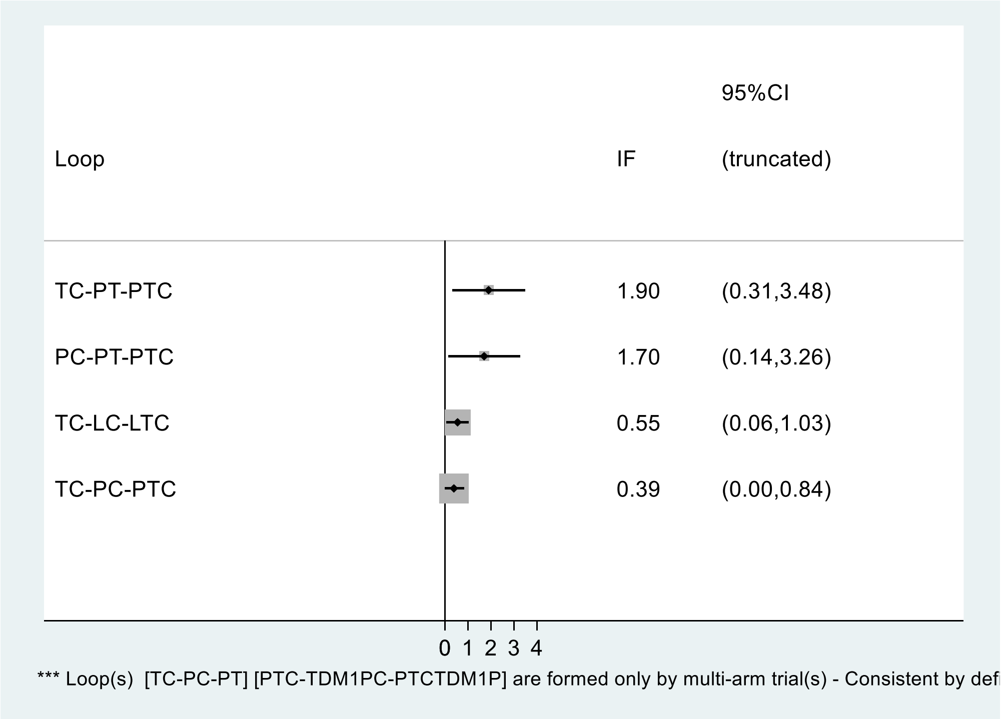

Figure S9. Inconsistency factor plot in network meta-analysis for serious adverse events (SAE). C = Chemotherapy; TC = Trastuzumab + chemotherapy; LTC = Lapatinib + trastuzumab +chemotherapy; LC = Lapatinib + chemotherapy.

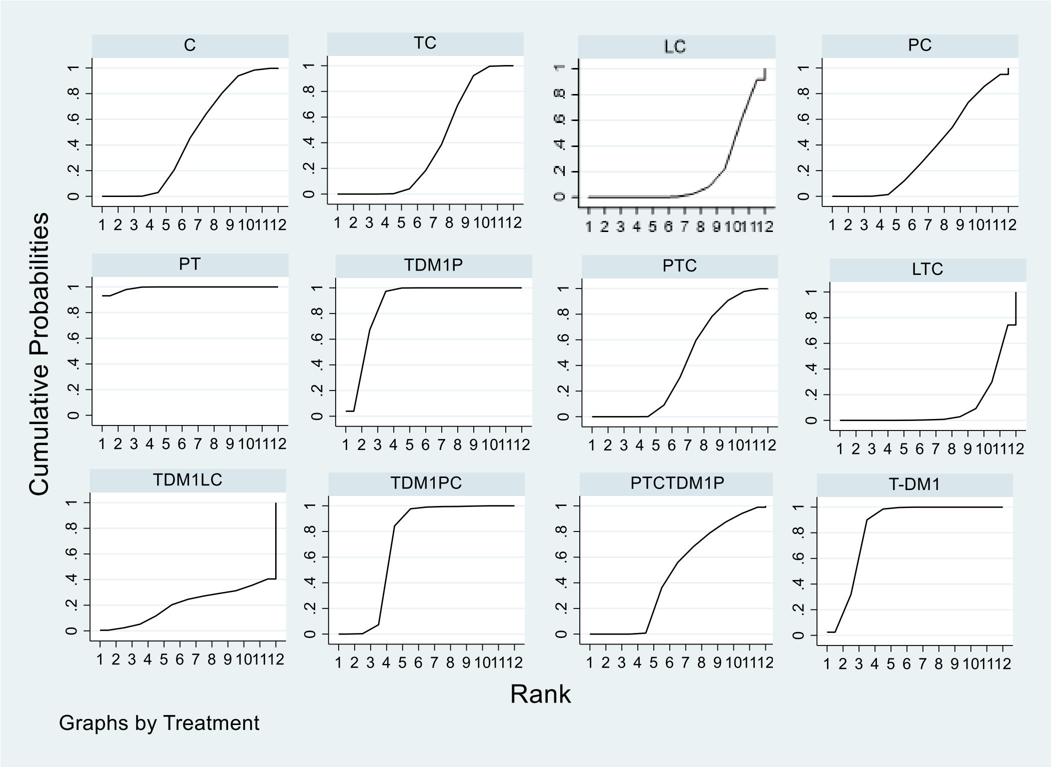

Treatment	SUCRA	PrBest	MeanRank
C	46	0	6.9
TC	38.4	0	7.8
PC	35.1	0	8.1
LC	16.8	0	10.2
PT	99.2	93.1	1.1
T-DM1P	88	3.9	2.3
PTC	42.4	0	7.3
LTC	10.7	0	10.8
T-DM1LC	20.8	0.5	9.7
T-DM1PC	71.6	0	4.1
PTC_T-DM1P	47.3	0	6.8
T-DM1	83.9	2.5	2.8

Figure S10. Surface Under the Cumulative Ranking Curves (SUCRA) for serious adverse events (SAE). C = Chemotherapy; TC = Trastuzumab + chemotherapy = PC = Pertuzumab + chemotherapy; LC = Lapatinib + chemotherapy; PTC = Pertuzumab + trastuzumab + chemotherapy; P = trastuzumab emtansine+ pertuzumab; T-DM1LC = trastuzumab emtansine +lapatnib + chemotherapy; T-DM1PC = trastuzumab emtansine +pertuzumab + chemotherapy; PTC_T-DM1P = pertuzumab + trastuzumab + chemotherapy followed by trastuzumab emtansine + pertuzumab; T-DM1 = Trastuzumab emtansine.

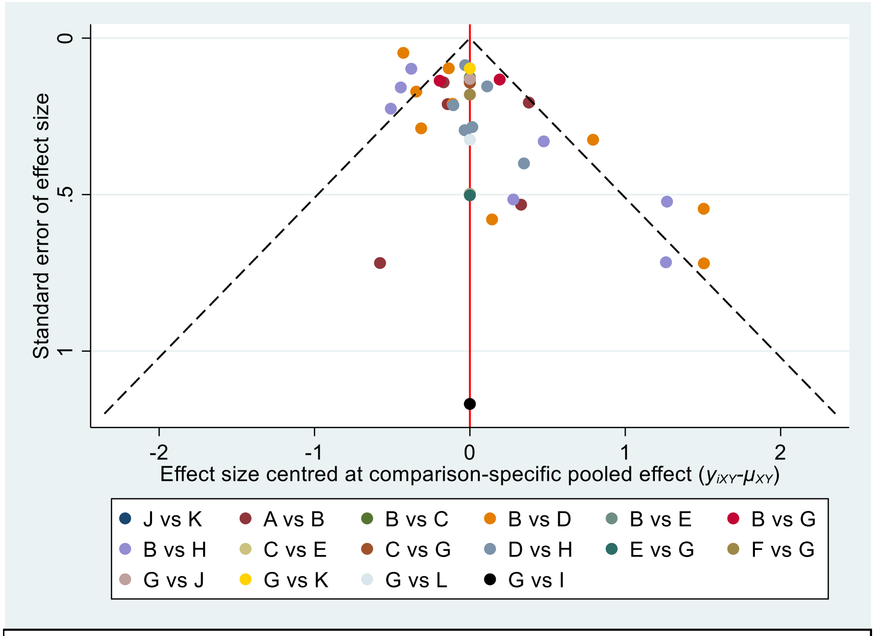

A: C (1), B: TC (2), C: PC (3), D: LC (4), E: PT (5), F: T-DM1P (6), G: PTC (7), H: LTC (8), I: T-DM1LC (9), J:T-DM1PC (10), K:PTC_T-DM1P (11), L:T-DM1 (12)

Figure S11. Comparison adjusted funnel plot of network meta-analysis for serious adverse events (SAE). C = Chemotherapy; TC = Trastuzumab + chemotherapy; LTC = Lapatinib + trastuzumab +chemotherapy; LC = Lapatinib + chemotherapy; T-DM1LC = trastuzumab emtansine + lapatnib + chemotherapy; T-DM1PC = Trastuzumab emtansine + pertuzumab + chemotherapy; T-DM1P = Trastuzumab emtansine + pertuzumab; PTC_T-DM1P = pertuzumab + trastuzumab + chemotherapy followed by trastuzumab emtansine + pertuzumab; T-DM1 = Trastuzumab emtansine.

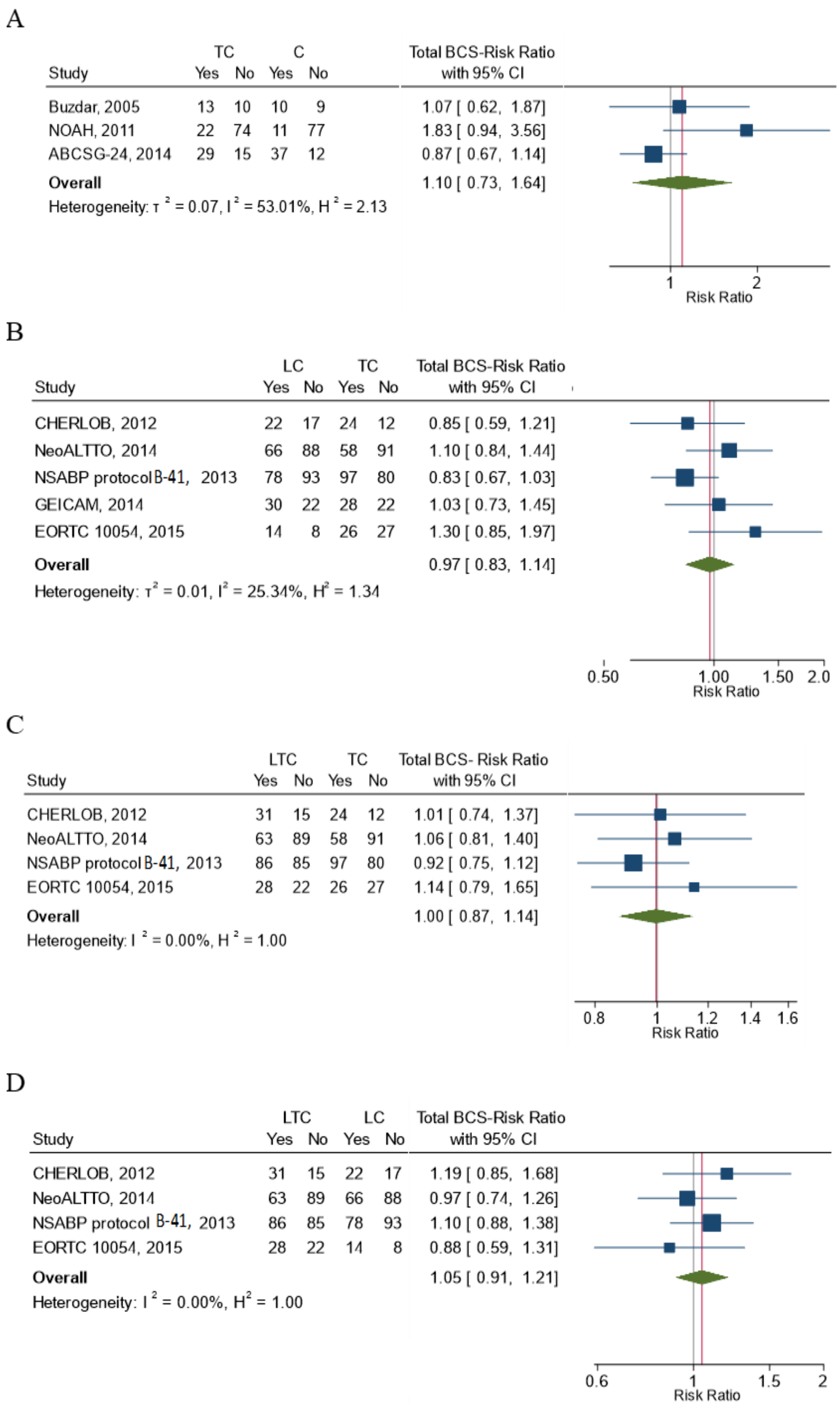

Figure S12. Forest plots of pairwise meta-analyses for total Breast Conservation Surgery. (**A**) TC vs C; (**B**) TC vs LC; (**C**) LTC vs TC; (**D**) LTC vs LC. C = Chemotherapy; TC = Trastuzumab + chemotherapy; LTC = Lapatinib + trastuzumab +chemotherapy; LC = Lapatinib + chemotherapy.

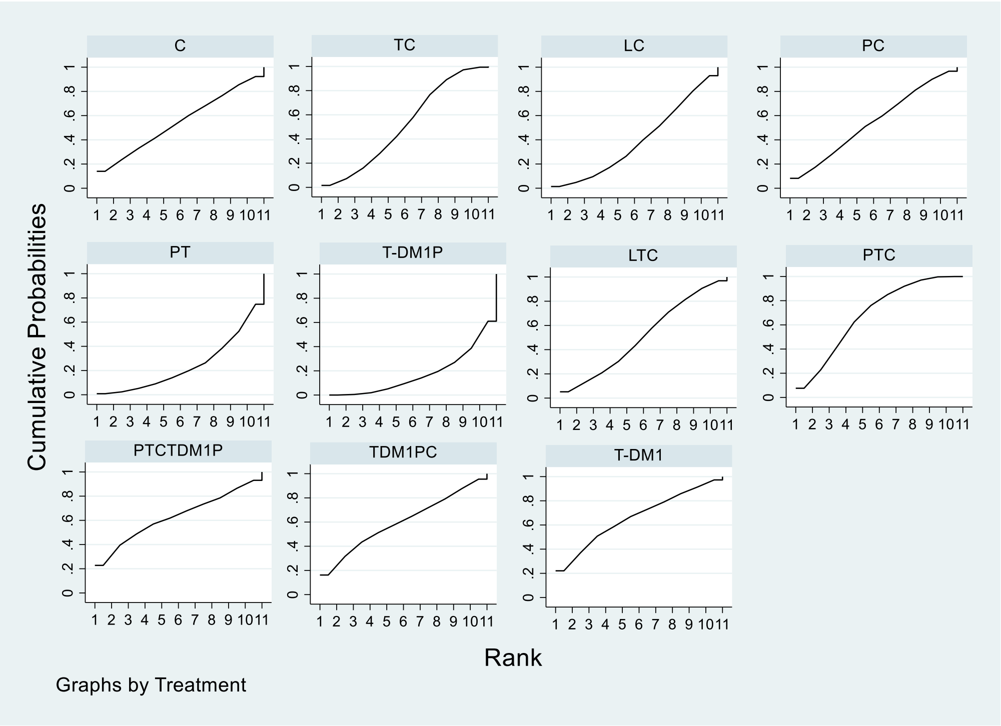

Treatment	SUCRA	PrBest	MeanRank
C	54.6	14	5.5
TC	51.5	1.6	5.8
PC	54.1	8.2	5.6
LC	38.9	1.4	7.1
PT	24.4	0.9	8.6
T-DM1P	17.7	0	9.2
PTC	68.5	7.6	4.1
LTC	51	5.2	5.9
T-DM1PC	60	16.2	5
PTC_T-DM1P	63	22.8	4.7
T-DM1	66.2	22.1	4.4

Figure S13. Surface under the cumulative ranking curves (SUCRA) of total breast conservation surgery (BCS). C = Chemotherapy; TC = Trastuzumab + chemotherapy; PC = Pertuzumab + chemotherapy; LC = Lapatinib + chemotherapy; PTC = Pertuzumab + trastuzumab + chemotherapy; LTC = Lapatinib + trastuzumab +chemotherapy; PT = Pertuzumab + trastuzumab; T-DM1P = trastuzumab emtansine+ pertuzumab; T-DM1PC = trastuzumab emtansine + pertuzumab + chemotherapy; PTC_T-DM1P = pertuzumab + trastuzumab + chemotherapy followed by trastuzumab emtansine + pertuzumab; T-DM1 = Trastuzumab emtansine.

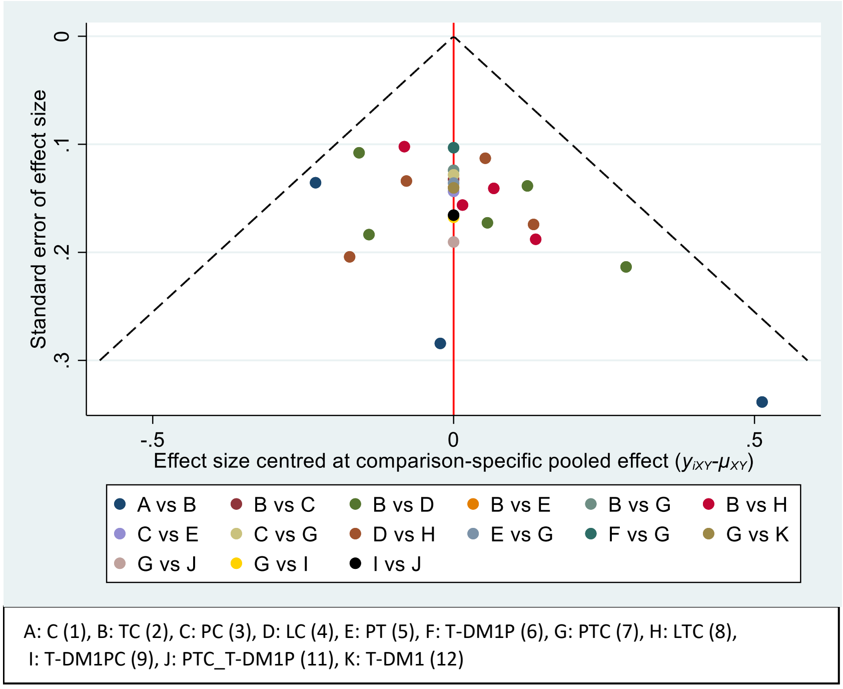

Figure S14. Comparison adjusted funnel plot of network meta-analysis for total Breast Conservation Surgery (BCS).

Table S1. Summary of studies and total number of patients included for each treatment regimen.

No.	Treatment Regimen	Regimen Abbreviation	Number of Trials	*n*
1	Chemotherapy only ©	C	5	259
2	Trastuzumab (T) + chemotherapy ©	TC	16	1439
3	Lapatinib (L) + chemotherapy ©	LC	9	884
4	Pertuzumab (P) + chemotherapy ©	PC	1	96
5	Pertuzumab (P) + Trastuzumab (T)	PT	2	199
6	Pertuzumab (P) + Trastuzumab (T) + chemotherapy ©	PTC	7	755
7	Lapatinib (L) + Trastuzumab (T) + chemotherapy ©	LTC	7	632
8	Trastuzumab emtansine (T-DM1) + Pertuzumab (P)	T-DM1P	1	223
9	Trastuzumab emtansine (T-DM1) + Lapatinib (L) + chemotherapy ©	T-DM1LC	1	14
10	Trastuzumab emtansine (T-DM1) + Pertuzumab (P) followed by response guided chemotherapy © or continued Trastuzumab emtansine (T-DM1) + Pertuzumab (P)	T-DM1PC	1	101
11	Pertuzumab (P)+ Trastuzumab (T) + chemotherapy (C) followed by Trastuzumab emtansine (T-DM1) + Pertuzumab (P)	PTC_T-DM1P	1	52
12	Trastuzumab emtansine (T-DM1)	T-DM1	1	99
	**Total patients**			4753

Table S2. Details of study's interventions.

Trial	Regimen	Anti-HER2	Neoadjuvant Anti HER2 Treatment		Neoadjuvant Chemotherapy Regimen		Use of Taxane/ Anthracycline
			Dosage	Duration (Weeks)	Chemotherapy	Dosage (Schedule) and Duration	
Buzdar et al. [29,30]	C	-	-	-	Paclitaxel and FECp (F: Fluorouracil, E: Epirubicin, Cp: Cyclophsphamide)	Paclitaxel 225 mg/m² 3 weekly 4 cycles, FECp (F 500 mg/m² IV on days 1 and 4, Cp 500 mg/m² IV on day 1, E 75 mg/m² on day 1 only) 3 weekly 4 cycles	Taxane, Anthracycline
	TC	T	T: 4 mg/kg IV loading dose then 2 mg/kg weekly	24 weeks	Paclitaxel and FECp	Same as above	Taxane, Anthracycline
CHERLOB	TC	T	T: 4 mg/kg IV loading dose then 2 mg/kg weekly	26 weeks	Paclitaxel and FECp	Paclitaxel 80 mg/m² weekly for 12 weeks then F 600 mg/m², E 75 mg/m², Cp 600 mg/m² 3 weekly 4 cycles	Taxane, Anthracycline
	LC	L	L: 1500 mg orally daily	26 weeks	Paclitaxel and FECp	Same as above	Taxane, Anthracycline
	LTC	L, T	T: 4 mg/kg IV loading dose then 2 mg/kg weekly, L: 1000 mg orally daily	26 weeks	Paclitaxel and FECp	Same as above	Taxane, Anthracycline
REMAGUS 02	C	-	-	-	Docetaxel and ECp	E 75 mg/m², Cp 750 mg/m² 3 weekly 4 cycles then docetaxel (100 mg/m²) 3 weekly 4 cycles	Taxane, Anthracycline
	TC	T	T: 8 mg/kg IV loading dose then 6 mg/kg 3 weekly	12 weeks 3 cycles	Docetaxel and ECp	Same as above	Taxane, Anthracycline
Chang et al. [34]	TC	T	T: 4 mg/kg IV loading dose then 2 mg/kg on Day 8, 15	12 weeks 4 cycles	Docetaxel and carboplatin	Docetaxel 75 mg/m² and carboplatin (AUC = 6) 3 weekly 4 cycles	Taxane
	C	-	-	-	Docetaxel and carboplatin	Same as above	Taxane
NOAH	TC	T	T: loading dose of 8 mg/ kg IV then 6 mg/kg 3 weekly	30 weeks (10 cycles)	Paclitaxel, Doxorubicin, FCp, methotrexate	Doxorubicin 60 mg/m² and paclitaxel 150 mg/m² 3 weekly 3 cycles, then paclitaxel 175 mg/m² 3 weekly 4 cycles. Cp 600 mg/m², methotrexate (40 mg/m²), and F 600	Taxane, Anthracycline

						mg/m²) on days 1 and 8 every 4 weeks 3 cycles.	
	C		-	-	Paclitaxel, Doxorubicin, FCp, Methotraxate	Same as above	Taxane, Anthracycline
NeoSphere	TC	T	T: 8 mg/kg IV loading dose, then 6 mg/kg 3 weekly.	12 weeks (4 cycles)	Docetaxel	Docetaxel 75 mg/m², escalating to 100 mg/m² (if tolerated) 3 weekly 4 cycles.	Taxane
	PTC	P, T	T: 8 mg/kg IV loading dose, then 6 mg/kg 3 weekly, P-loading dose 840 mg IV, then 420 mg 3 weekly.	12 weeks (4 cycles)	Docetaxel	Same as above	Taxane
	PT	P, T	T: 8 mg/kg IV loading dose, then 6 mg/kg 3 weekly, P: loading dose 840 mg IV, then 420 mg 3 weekly.	12 weeks (4 cycles)	-	-	-
	PC	P	P: loading dose 840 mg IV, then 420 mg 3 weekly.	12 weeks (4 cycles)	Docetaxel	Docetaxel 75 mg/m², escalating to 100 mg/m² (if tolerated) 3 weekly 4 cycles.	Taxane
GeparQuinto, GBG 44	TC	T	T: loading dose of 8 mg/kg IV then 6 mg/kg 3 weekly.	24 weeks (8 cycles)	Docetaxel and ECp	E 90 mg/m², Cp 600 mg/m² 3 weekly 4 cycles, then docetaxel 100 mg/m², 3 weekly 4 cycles.	Taxane, Anthracycline
	LC	L	L: 1250 mg orally daily	24 weeks	Docetaxel and ECp	Same as above	Taxane, Anthracycline
NeoALTTO	LC	L	L: 1500 mg orally daily	18 weeks (6 weeks then 12 weeks with C)	Paclitaxel	Paclitaxel 80 mg/m² weekly for 12 weeks.	Taxane
	TC	T	T: 4 mg/kg loading dose IV then 2 mg/kg weekly	18 weeks (6 weeks then 12 weeks with C)	Paclitaxel	Paclitaxel 80 mg/m² weekly for 12 weeks.	Taxane
	LTC	L, T	L: 1000 mg orally daily, T - 4 mg/kg IV loading dose then 2 mg/kg weekly.	18 weeks (6 weeks then 12 weeks with C)	Paclitaxel	Paclitaxel 80 mg/m² weekly 12 weeks.	Taxane
NSABP protocol B-41	TC	T	T: loading dose 4 mg/kg IV then 2 mg/kg weekly	12 weeks	Paclitaxel and Doxorubicin, Cp	Doxorubicin 60 mg/m² and Cp-600 mg/m² 3 weekly 4 cycles, then paclitaxel 80 mg/m² 4 weekly 4 cycles.	Taxane, Anthracycline

	LC	L	L: 1500 mg (reduced to 1250 mg) orally daily	12 weeks	Paclitaxel and Doxorubicin, Cp	Doxorubicin 60 mg/m² and Cp 600 mg/m² 3 weekly 4 cycles, then paclitaxel 80 mg/m² 4 weekly 4 cycles	Taxane, Anthracycline
	LTC	L, T	T: loading dose 4 mg/kg then 2 mg/kg IV weekly, L: 1000 mg (reduced to 750 mg) orally daily	12 weeks	Paclitaxel and Doxorubicin, Cp	Same as above	Taxane, Anthracycline
LPT109096	TC	T	T: 4 mg/kg loading dose then 2 mg/kg IV weekly	26 weeks (14 days prior to C)	Paclitaxel and FECp	F 500 mg/m², E 75 mg/m², Cp 500 mg/m², 3 weekly 4 cycles, then paclitaxel 80 mg/m² for 12 weeks	Taxane, Anthracycline
	LC	L	L: 1500 mg daily orally, (reduced to 1250 mg due to diarrhea)	26 weeks (14 days prior to C)	Paclitaxel and FECp	F 500 mg/m², E 75 mg/m², Cp 500 mg/m², 3 weekly 4 cycles, then paclitaxel 80 mg/m² for 12 weeks	Taxane, Anthracycline
	LTC	L, T	T: 4 mg/kg IV loading dose then 2 mg/kg weekly, L: 1000 mg orally daily. (reduced to 750 mg with FECp, then 1000 mg with paclitaxel).	26 weeks (14 days prior to C)	Paclitaxel and FECp	F 500 mg/m², E 75 mg/m², Cp 500 mg/m², 3 weekly 4 cycles, then paclitaxel 80 mg/m² for 12 weeks	Taxane, Anthracycline
ABCSG-24	C	-	-	-	Docetaxel, Epirubicin +/− carboplatin	E 75 mg/m², Docetaxel 75 mg/m² 3 weekly ± Carboplatin 1000 mg/m² orally, twice daily, days 1–13.	Taxane, Anthracycline
	TC	T	T: 8 mg/kg loading dose then 6 mg/kg IV, 3 weekly	18 weeks (6 cycles)	Docetaxel, Epirubicin +/− carboplatin	E 75 mg/m², Docetaxel 75 mg/m² 3 weekly ± Carboplatin 1000 mg/m² orally, twice daily, days 1–13.	Taxane, Anthracycline
GEICAM/2006-14	TC	T	T: loading dose of 8 mg/kg then 6 mg/kg IV 3 weekly	12 weeks (4 cycles)	Docetaxel, epirubucin	E 90 mg/m², Cp 600 mg/m² 3 weekly 4 cycles then docetaxel 100 mg/m², 3 weekly 4 cycles	Taxane, Anthracycline
	LC	L	L: 1250 mg orally daily	12 weeks	Docetaxel, epirubucin	E 90 mg/m², Cp 600 mg/m², 3 weekly 4 cycles then docetaxel 100 mg/m², 3 weekly 4 cycles	Taxane, Anthracycline
EORTC 10054	LC	L	L: 1000 mg orally daily	18 weeks	Docetaxel and FECp	Docetaxel 100 mg/m² 3 weekly 3 cycles then FECp (F 500 mg/m², E	Taxane, Anthracycline

						100 mg/m², Cp 500 mg/m²), 3 weekly 3 cycles	
	TC	T	T: 4 mg/kg loading dose then 2 mg/kg IV weekly	18 weeks	Docetaxel and FECp	Same as above	Taxane, Anthracycline
	LTC	L, T	T: 4 mg/kg loading dose then 2 mg/kg IV weekly, L: 1000 mg orally daily	18 weeks	Docetaxel and FECp	Same as above	Taxane, Anthracycline
CALGB 40601	LTC	L, T	T: loading dose of 4 mg/kg IV then 2 mg/kg IV weekly, L: 1000 mg orally daily	16 weeks	Paclitaxel	Paclitaxel 80 mg/m² weekly for 16 weeks	Taxane
	TC	T	T: loading dose of 4 mg/kg IV then 2 mg/kg weekly IV	16 weeks	Paclitaxel	Paclitaxel 80 mg/m² weekly for 16 weeks	Taxane
	LC	L	L: 1500 mg orally daily	16 weeks	Paclitaxel	Paclitaxel 80 mg/m² weekly for 16 weeks	Taxane
WSG-ADAPT	PT	P, T	T: 8 mg/kg loading dose then 6 mg/kg, IV 3 weekly, P: loading dose 840 mg IV then by 420 mg	12 weeks (4 cycles)	-	-	
	PTC	P, T	T- 8 mg/kg IV loading dose then 6 mg/kg, P- loading dose 840 mg then 420 mg IV, 3 weekly	12 weeks (4 cycles)	Paclitaxel	Paclitaxel 80 mg/m² every 3 weekly 12 weeks	Taxane
KRISTINE	T-DM1 P	T-DM1, P	T-DM1: 3·6 mg/kg IV, P: loading dose 840 mg then 420 mg IV 3 weekly	18 weeks (6 cycles)	-	-	
	PTC	P, T	T: loading dose 8 mg/kg then 6 mg/kg IV, P: loading dose 840 mg given then 420 mg IV 3 weekly.	18 weeks (6 cycles)	Docetaxel and carboplatin	Docetaxel 75 mg/m², carboplatin [AUC] 6 mg, 3 weekly 6 cycles	Taxane
Teal study	T-DM1 LC	T-DM1, L	T-DM1: 3.0 mg/kg IV, 3 weekly, L: 750 mg orally daily	18 weeks (6 weeks, then 12 weeks with C)	Paclitaxel	Nab-paclitaxel 80 mg/m² weekly 12 weekly	Taxane

	PTC	P, T	T: 4 mg/kg loading dose then 2 mg/kg IV weekly, P: 840 mg loading dose then 420 mg 3 IV weekly.	18 weeks (6 weeks, then 12 weeks with C)	Paclitaxel	Paclitaxel 80 mg/m² weekly for 12 weeks then FECp (F 600 mg/m², E-75 mg/m², and Cp 600 mg/m²), 3 weekly 4 cycles	Taxane
PEONY	PTC	P, T	T: 8 mg/kg loading dose then 6 mg/kg IV, P: 840 mg loading dose then 420 mg 3 weekly IV	12 weeks (4 cycles)	Docetaxel	Docetaxel 75 mg/m², 3 weekly 12 weeks	Taxane
	TC	T	T: 8 mg/kg loading dose then 6 mg/kg IV, 3 weekly	12 weeks (4 cycles)	Docetaxel	Same as above	Taxane
Masuda et al. [11]	PTC	P, T	T: 8 mg/kg loading dose then 6 mg/kg IV, P: 840 mg loading dose then 420 mg IV.	18 weeks (6 cycles)	Docetaxel	Docetaxel 75 mg/m², Carboplatin AUC 6 mg 3 weekly 6 cycles	Taxane
	PTC_ T-DM1P	P, T, T-DM1, P	T: 8 mg/kg loading dose then 6 mg/kg IV, P: 840 mg loading dose then 420 mg IV 3 weekly (1–4 cycles), and P: 420 mg IV, T-DM1 3.6 mg/kg IV 3 weekly; 5- 8 cycles	24 weeks (8 cycles)	Docetaxel	Docetaxel 75 mg/m², Carboplatin AUC 6 mg 3 weekly 1–4 cycles	Taxane
	T-DM1 PC	T-DM1, P	P: loading dose 840 mg then 420 mg IV, T-DM1: 3.6 mg/kg IV 3 weekly (1–4 cycles), For Responders continued T-DM1P (5–6 cycles) (response—reduction of tumour size in MRI/ reduced Ki67 level at 4 cycles)	12 weeks (4 cycles) then another 2 cycles (6 weeks) if response or C for another 12 weeks (4 cycles)	FECp	For non-responders: FECp F 500 mg/m²; E 100 mg/m2; Cp 500 mg/m² (Dose reduction of E, 75 or 60 mg/m² or discontinued, if AE) 3 weekly 5–8 cycles	Anthracycline
TRIO-US B07	TC	T	T: Run in cycle 8 mg/kg IV (cycle 1), followed by 6 mg/kg (6 cycles)	21 weeks (loading + 3 weekly 6 cycles with C) (7 cycles)	Docetaxel and carboplatin	Docetaxel 75 mg/m², Carboplatin AUC 6 mg 3 weekly 6 cycles	Taxane

	LC	L	L: Run in cycle 1000 mg daily orally for 21 days, followed by 1000 mg/day orally daily	21 weeks (loading 21 days + daily 18 weeks with C)	Docetaxel and carboplatin	Docetaxel 75 mg/m², Carboplatin AUC 6 mg 3 weekly 6 cycles	Taxane
	LTC	L, T	T: Run in cycle 8 mg/kg IV once and followed by 6 mg/kg 6 cycles; L: Run in cycle 1000 mg daily orally for 21 days, followed by 1000 mg orally daily 18 weeks	21 weeks (loading 1 cycle + 3 weekly 6 cycles with C) (7 cycles)	Docetaxel and carboplatin	Docetaxel 75 mg/m², Carboplatin AUC 6 mg 3 weekly 6 cycles	Taxane
Hatschek et al. [54]	PTC	P, T	T: Subcutaneous 600 mg; P: loading dose 840 mg then 420 mg 3 weekly	18 weeks (6 cycles) if no response/toxicity after 2 cycles switched to T-DM1 regimen 4 cycles	Docetaxel	Docetaxel 75 mg/m² first dose, then 100 mg/m² 3 weekly	Taxane
	T-DM1	-	T-DM1 (3.6 mg/kg) 3 weekly	18 weeks (6 cycles) If no response/toxicity after 2 cycles switched to PTC regimen 4 cycles	-	-	-

C = Chemotherapy; TC, Trastuzumab + chemotherapy; PC = Pertuzumab + chemotherapy; LC = Lapatinib + chemotherapy; PTC = Pertuzumab + trastuzumab + chemotherapy; LTC = Lapatinib + trastuzumab +chemotherapy; PT = Pertuzumab + trastuzumab; T-DM1P = T-DM1+ pertuzumab; T-DM1LC = T-DM1 + lapatinib + chemotherapy; T-DM1PC = T-DM1 + Pertuzumab followed by response chemotherapy or guided continued T-DM1 + pertuzumab; PTC_T-DM1P = Pertuzumab + Trastuzumab + chemotherapy followed by T-DM1 + Pertuzumab; T-DM1 = trastuzumab emtansine; T = Trastuzumab; P = Pertuzumab; L = Lapatinib; F = Fluorouracil; E = Epirubicin; Cp = Cyclophsphamide; IV- Intravenous; AUC = area under the plasma concentration-time curve.

Table S3. Results of risk of bias assessment.

Reference	Regimens	Outcome	Randomization Process	Deviations from Intended Interventions	Missing Outcome Data	Measurement of the Outcome	Selection of the Reported Result	Overall Bias
Buzdar et al. [29,30]	TC, C	pCR	Low	Low	Low	Low	Low	Low
		SAE	Low	Low	High	Low	Some concerns	High
		BCS	Low	Low	Low	Low	Low	Low
CHERLOB	TC, LC, LTC	pCR	Low	Some concerns	Low	Low	Low	Some concerns
		SAE	Low	Some concerns	Low	Low	Some concerns	Some concerns
		BCS	Low	Some concerns	Low	Low	Low	Some concerns
		DFS	Low	Some concerns	Some concerns	Low	Low	Some concerns
RAMAGUS 02	C, TC	pCR	Low	Low	Low	Low	Low	Low
		SAE	Low	Low	Low	Low	Low	Low
Chang et al. [34]	C, TC	pCR	Some concerns	Low	Some concerns	Some concerns	Low	Some concerns
		SAE	Some concerns	Low	Some concerns	Some concerns	Low	Some concerns
NOAH	C, TC	pCR	Low	Low	Low	Low	Low	Low
		SAE	Low	Low	Low	Low	Some concerns	Some concerns
		BCS	Low	Low	Low	Low	Low	Low
		DFS	Low	Low	Low	Low	Low	Low
NeoSphere	TC, PT, PTC, PC	pCR	Low	Low	Low	Low	Low	Low
		SAE	Low	Low	Low	Low	Some concerns	Low
		BCS	Low	Low	Some concerns	Low	Low	Some concerns
		DFS	Low	Low	Low	Low	Low	Low
GeparQuinto	TC, LC	pCR	Low	Low	Low	Low	Low	Low
		SAE	Low	Low	Low	Low	Some concerns	Low

		DFS	Low	Low	Low	Low	Low	Low
NeoALTTO	TC, LC, LTC	pCR	Low	Low	Some concerns	Low	Low	Some concerns
		SAE	Low	Low	Some concerns	Low	Some concerns	Some concerns
		BCS	Low	Low	Low	Low	Low	Low
		DFS	Low	Low	Some concerns	Low	Low	Some concerns
NSABP Protocol B-41	TC, LC, LTC	pCR	Low	Low	Low	Low	Low	Low
		SAE	Low	Low	Some concerns	Low	Low	Some concerns
		BCS	Low	Low	Low	Low	Low	Low
LPT109096	TC, LC, LTC	pCR	Some concerns	Low	Some concerns	Low	Low	Some concerns
		SAE	Some concerns	Low	Some concerns	Low	Low	Some concerns
ABCSG-24	C, TC	pCR	Low	Low	Low	Low	Low	Low
		SAE	Low	Low	Low	Low	Low	Low
		BCS	Low	Low	Low	Low	Low	Low
GEICAM trial	TC, LC	pCR	Low	Low	Low	Low	Low	Low
		SAE	Low	Low	Low	Low	Low	Low
		BCS	Low	Low	Low	Low	Low	Low
EORTC 10054	TC, LC, LTC	pCR	Low	Some concerns	Some concerns	Low	Low	Some concerns
		SAE	Low	Some concerns	Some concerns	Low	Some concerns	Some concerns
		BCS	Low	Some concerns	Low	Low	Low	Some concerns
CALGB 40601	TC, LC, LTC	pCR	Low	Low	Low	Low	Low	Low
		SAE	Low	Low	Some concerns	Low	Low	Some concerns
WSG-ADAPT	PT, PTC	pCR	Some concerns	Low	Some concerns	Low	Low	Some concerns
		SAE	Some concerns	Low	Some concerns	Low	High	Some concerns
		BCS	Some concerns	Low	Low	Low	Low	Some concerns

KRISTINE	PTC, T-DM1P	pCR	Low	Low	Low	Low	Low	Low
		SAE	Low	Low	Some concerns	Low	Low	Some concerns
		BCS	Low	Low	Low	Low	Low	Low
		DFS	Low	Low	Low	Low	Low	Low
Teal study	PTC, T-DM1LC	pCR	Low	Low	Low	Low	Low	Low
		SAE	Low	Low	Low	Low	Some concerns	Low
PEONY	TC, PTC	pCR	Low	Low	Low	Low	Low	Low
		SAE	Low	Low	Low	Low	Low	Low
Masuda et al. [11]	PTC, PTC_T-DM1P, T-DM1PC	pCR	Low	Low	Low	Low	Low	Low
		SAE	Low	Low	Low	Low	Low	Low
		BCS	Low	Low	Low	Low	Low	Low
TRIO-US	TC, LC, LTC	pCR	Low	Some concerns	Low	Low	Low	Some concerns
		SAE	Low	Some concerns	Some concerns	Low	Low	Some concerns
Hatschek et al. [54]	PTC, T-DM1	pCR	Low	Some concerns	Low	Low	Low	Some concerns
		SAE	Low	Some concerns	Low	Low	Low	Some concerns
		BCS	Low	Low	Low	Low	Low	Low
		DFS	Low	Some concerns	Low	Low	Low	Some concerns

C = Chemotherapy; TC = Trastuzumab + chemotherapy, PC = Pertuzumab + chemotherapy; LC = Lapatinib + chemotherapy; PTC = Pertuzumab + trastuzumab + chemotherapy; LTC = Lapatinib + trastuzumab +chemotherapy; PT = Pertuzumab + trastuzumab; T-DM1P = trastuzumab emtansine + pertuzumab; T-DM1LC = trastuzumab emtansine +lapatinib + chemotherapy; T-DM1PC = trastuzumab emtansine + pertuzumab + chemotherapy; PTC_T-DM1P = pertuzumab + trastuzumab + chemotherapy followed by trastuzumab emtansine + pertuzumab, T-DM1= Trastuzumab emtansine.

Table S4. Results of meta regression of LTC vs LC and LC vs TC for pathologic complete response (pCR) outcome.

(A) LTC vs LC			
**Co-variables**	**Number of trials**	**I^2 (%)**	**Tau^2**
Null model	7	42.12	0.0339
Duration of anti HER2 treatment	7	34.95	0.03033
Chemotherapy regimen type	7	0.00	0
Median age	6	0.00	0
HR negative percentage (%HR−ve)	7	40.84	0.03751
Nodal positive percentage	3	0.00	0
T3-T4 tumours percentage (%T3-T4)	4	41.85	0.03902
**(B) LC vs TC**			
Co-variables	Number of trials	I^2 (%)	Tau^2
Null model	9	25.96	0.01439
Duration of anti HER2 treatment	9	22.01	0.01545
Chemotherapy regimen type	9	24.30	0.01348
Median age	8	0.00	0
HR negative percentage (%HR−ve)	8	23.62	0.01254
Nodal positive percentage	5	0.00	0
T3-T4 tumours percentage (%T3-T4)	6	0.00	0

C = Chemotherapy; TC = Trastuzumab + chemotherapy; LTC = Lapatinib + trastuzumab +chemotherapy; LC = Lapatinib + chemotherapy.

Table S5. Subgroup analyses of LTC vs LC for pathological complete response (pCR) outcome.

Subgroup	No. of Trials	pCR-RR (95% CI)	I^2	Tau^2
Duration of anti HER2 therapy				
18 weeks or less	4	1.68 (95% CI: 1.21–2.33)	66.67	0.070
More than 18 weeks	3	1.67 (95% CI: 1.33–2.07)	0.00	0.000
HR−ve Percentage (%HR−ve)				
<45%HR−ve	4	1.58 (95% CI: 1.21–2.08)	38.89	0.030
>45%HR−ve	3	1.77 (95% CI: 1.24–2.53)	35.94	0.036
Chemotherapy type				
Taxane	3	2.13 (95% CI: 1.65–2.75)	0.00	0.000
Taxane + Anthracycline	4	1.33 (95% CI: 1.12–1.58)	0.00	0.000
Overall	7	1.66 (95% CI: 1.33–2.07)	42.12	0.034

RR = Risk ratio; LTC = Lapatinib + trastuzumab +chemotherapy; LC = Lapatinib + chemotherapy.

Table S6. Subgroup analyses of LC vs TC for pathological complete response (pCR) outcome.

**Subgroup**	**No. of trials**	**pCR-RR (95% CI)**	**I^2**	**Tau^2**
Duration of anti HER2 therapy				
18 weeks or less	5	0.74 (95% CI: 0.57–0.95)	43.85%	0.035
More than 18 weeks	4	0.70 (95% CI: 0.58–0.84)	0%	0.000
HR−ve Percentage (%HR−ve)				
<45%HR−ve	6	0.75 (95% CI: 0.61–0.92)	40.59%	0.024
>45%HR−ve	3	0.70 (95%CI: 0.52–0.94)	0.79%	0.001
Chemotherapy type				
Taxane	3	0.65 (95% CI: 0.49–0.85)	0%	0.000
Taxane + Anthracycline	6	0.78 (95% CI: 0.63–0.97)	41.68%	0.026
Overall	9	0.74 (95%CI: 0.63–0.87)	25.96%	0.014
Median age				
50 years or more	4	0.69 (95% CI: 0.59–0.82)	0%	0.000
<50 years	4	0.63 (95% CI: 0.45–0.87)	0%	0.000
Overall	8	0.68 (95% CI:0.58 - 0.79)	0%	0.000
T3-T4 percentage (%T3-T4)				
<40% T3–4	2	0.65 (95%CI: 0.54–0.80)	0%	0.000
>40% T3–4	4	0.89 (95% CI: 0.74–1.06)	0%	0.000
Overall	6	0.76 (95% CI: 0.63–0.92)	39.19%	0.02
Nodal positivity (%)				
65% or more	3	0.66 (95% CI: 0.54–0.79)	0%	0.000
<65%	2	0.96 (95% CI: 0.78–1.18)	0%	0.000
Overall	5	0.76 (95% CI: 0.60–0.96)	50.71%	0.032

RR = Risk ratio; LTC = Lapatinib + trastuzumab +chemotherapy; TC = Trastuzumab + chemotherapy.

Table S7. Treatment comparisons and data used for pooling pathological complete response (pCR) outcome in network meta-analysis.

Trial	Year	Regimen	Total Number	Number achieving pCR
Buzdar et al. [29,30]	2005	C	19	5
		TC	23	15
CHERLOB	2012	TC	36	9
		LC	39	10
		LTC	46	21
REMAGUS 02	2017	C	58	11
		TC	62	16
Chang et al. [34]	2010	TC	15	6
		C	15	1
NOAH	2011	TC	117	45
		C	118	23
NeoSphere	2012	TC	107	23
		PTC	107	42
		PT	107	12
		PC	96	17
GeparQuinto	2012	TC	307	137
		LC	308	93
NeoALTTO	2012	LC	154	30
		TC	149	40
		LTC	152	68
NSABP protocol B-41	2013	TC	181	87
		LC	174	81
		LTC	174	103
LPT109096	2013	TC	33	14
		LC	34	13
		LTC	33	17
ABCSG-24	2014	C	49	13
		TC	44	17
GEICAM	2014	TC	50	23
		LC	52	12
EORTC 10054	2015	LC	23	8
		TC	53	27
		LTC	52	27
CALGB 40601	2016	LTC	117	60
		TC	118	51
		LC	64	17
WSG-ADAPT	2017	PT	92	31
		PTC	42	38
KRISTINE	2018	T-DM1P	223	99
		PTC	221	123
Teal	2019	T-DM1LC	14	12
		PTC	16	10
PEONY	2019	PTC	219	86
		TC	110	24
Masuda et al. [11]	2020	PTC	51	29
		PTC_T-DM1P	52	37
		T-DM1PC	101	58
TRIO-US B07	2020	TC	34	16
		LC	36	9
		LTC	58	30
Hatschek et al. [54]	2021	PTC	99	45
		T-DM1	99	43

C = Chemotherapy; TC = Trastuzumab + chemotherapy, PC = Pertuzumab + chemotherapy; LC = Lapatinib + chemotherapy; PTC = Pertuzumab + trastuzumab + chemotherapy; LTC = Lapatinib + trastuzumab

+chemotherapy; PT = Pertuzumab + trastuzumab; T-DM1P = trastuzumab emtansine + pertuzumab; T-DM1LC = trastuzumab emtansine +lapatinib + chemotherapy; T-DM1PC = trastuzumab emtansine + pertuzumab followed by response guided chemotherapy or continued trastuzumab emtansine + Pertuzumab; PTC_T-DM1P = pertuzumab + trastuzumab + chemotherapy followed by trastuzumab emtansine + pertuzumab; T-DM1 = Trasuzumab emtansine.

Table S8. Results of meta regression of LC vs TC and LTC vs TC comparisons for serious adverse events (SAE) outcome.

**(A) LC vs TC**			
**Co-variables**	**Number of trials**	**I^2 (%)**	**Tau^2**
Null model	9	79.97	0.1034
Duration of anti HER2 treatment	9	75.82	0.1433
Chemotherapy regimen type	9	62.33	0.0394
Median age	8	82.06	0.2116
HR negative percentage (%HR−ve)	8	84.20	0.1440
T3-T4 tumours percentage (%T3-T4)	6	62.84	0.0618
**(B) LTC vs TC**			
**Co-variables**	**Number of trials**	**I^2 (%)**	**Tau^2**
Null model	7	73.78	0.1512
Duration of anti HER2 treatment	7	78.08	0.3457
Chemotherapy regimen type	7	36.71	0.0294
Median age	6	81.11	0.6528
HR negative percentage (%HR−ve)	7	78.01	0.3386
T3-T4 tumours percentage (%T3-T4)	4	70.92	0.1065

C = Chemotherapy; TC = Trastuzumab + chemotherapy; LTC = Lapatinib + trastuzumab +chemotherapy; LC = Lapatinib + chemotherapy.

Table S9. Treatment comparisons and data used for pooling Serious adverse events (SAE) outcome in network meta-analysis.

Trial	Year	Regimen	Total Number	Number with SAE
Buzdar et al. [29,30]	2005	C	19	11
		TC	23	21
CHERLOB	2012	TC	36	2
		LC	39	14
		LTC	46	16
REMAGUS 02	2017	C	58	38
		TC	62	37
Chang et al. [34]	2010	TC	15	6
		C	15	4
NOAH	2011	TC	117	3
		C	118	5
NeoSphere	2012	TC	107	61
		PTC	107	48
		PT	107	4
		PC	96	52
GeparQuinto	2012	TC	307	237
		LC	308	222
NeoALTTO	2012	LC	154	36
		TC	149	11
		LTC	152	32
NSABP protocol B-41	2013	TC	181	89
		LC	174	107
		LTC	174	104
LPT109096	2013	TC	33	22
		LC	34	23
		LTC	33	25
ABCSG-24	2014	C	49	25
		TC	44	21
GEICAM	2014	TC	50	21
		LC	52	28
EORTC 10054	2015	LC	23	10
		TC	53	22
		LTC	52	23
CALGB 40601	2016	LTC	117	25
		TC	118	4
		LC	64	14
WSG-ADAPT	2017	PT	92	5
		PTC	42	5
KRISTINE	2018	T-DM1P	223	29
		PTC	221	141
Teal	2019	T-DM1LC	14	2
		PTC	16	1
PEONY	2019	PTC	219	106
		TC	110	46
Masuda et al. [11]	2020	PTC	51	43
		PTC_T-DM1P	52	40
		T-DM1PC	101	43
TRIO-US B07	2020	TC	34	4
		LC	36	7
		LTC	58	16
Hatschek et al. [54]	2021	PTC	99	7
		T-DM1	99	12

C = Chemotherapy; TC = Trastuzumab + chemotherapy, PC = Pertuzumab + chemotherapy; LC = Lapatinib + chemotherapy; PTC = Pertuzumab + trastuzumab + chemotherapy; LTC = Lapatinib + trastuzumab

+chemotherapy; PT = Pertuzumab + trastuzumab; T-DM1P = trastuzumab emtansine + pertuzumab; T-DM1LC = trastuzumab emtansine +lapatinib + chemotherapy; T-DM1PC = trastuzumab emtansine + pertuzumab followed by response guided chemotherapy or continued trastuzumab emtansine + Pertuzumab; PTC_T-DM1P = pertuzumab + trastuzumab + chemotherapy followed by trastuzumab emtansine + pertuzumab.

Table S10. Comparison of study characteristics in treatment loop with inconsistency for serious adverse events (SAE) outcome.

Trial	Regimen	Chemotherapy Type	Duration of Treatment (Weeks)	Median Age (Years)	Tumour Size		% HR Positive	% HR Negative	% Node Negative (N0)	% Node Positive	ECOG Performance Status ≤1 (%)
					Median Size of Tumour (mm)	T Stage (%)					
NeoSphere (Gianni, et al., 2012) [38]	TC	Taxane (Docetaxel)	12	50	50 (20–200)	T2–3, T4a–c (94%), T4d (7%)	47	53	30	70	100
	PTC	Taxane (Docetaxel)		50	55 (20–150)	T2–3, T4a–c (91%), T4d (9%)	47	53	29	71	100
	PT	-		49	50 (20–200)	T2–3, T4a–c (94%), T4d (7%)	48	52	30	70	100
	PC	Taxane (Docetaxel)		49	50 (0–180)	T2–3, T4a–c (95%), T4d (5%)	48	52	29	71	100
WSG-ADAPT (Nitz et al., 2017) [49]	PT	-	12	54	-	T1 (41%), T2–3 (58%), T4 (1%)	0	100	54.4	45.6	100
	PTC	Taxane (Paclitaxel)		51.5	-	T1 (41%), T2–3-(59%)	0	100	61.9	38.1	100
PEONY (Shao et al., 2019) [51]	PTC	Taxane (Docetaxel)	12	49	-	T2 (71%), T3 (20%), T4 (9%)	52	48	73	27	100
	TC	Taxane (Docetaxel)		49	-	T2 (65%), T3 (26%), T4 (9%)	51	49	81	19	100

C = Chemotherapy; TC = Trastuzumab + chemotherapy, PC = Pertuzumab + chemotherapy; PTC = Pertuzumab + trastuzumab + chemotherapy; PT = Pertuzumab + trastuzumab.

Table S11. Risk ratios and 95% confidence intervals of network meta-analysis of total breast conservative surgery (BCS) outcome.

C	0.99 (0.77,1.26)	1.00 (0.70,1.43)	0.95 (0.71,1.27)	0.88 (0.61,1.27)	0.84 (0.56,1.26)	1.06 (0.75,1.50)	0.99 (0.74,1.32)	1.04 (0.64,1.68)	1.06 (0.63,1.77)	1.07 (0.69,1.67)
-	**TC**	1.01 (0.78,1.32)	0.96 (0.83,1.11)	0.89 (0.67,1.18)	0.85 (0.62,1.18)	1.07 (0.84,1.38)	1.00 (0.87,1.15)	1.05 (0.70,1.60)	1.07 (0.68,1.68)	1.09 (0.75,1.58)
-	-	**PC**	0.95 (0.70,1.29)	0.88 (0.66,1.17)	0.84 (0.61,1.17)	1.06 (0.82,1.37)	0.99 (0.73,1.33)	1.04 (0.68,1.59)	1.06 (0.67,1.67)	1.07 (0.73,1.57)
-	-	-	**LC**	0.92 (0.68,1.26)	0.89 (0.62,1.26)	1.12 (0.84,1.49)	1.04 (0.90,1.20)	1.09 (0.71,1.70)	1.11 (0.69,1.79)	1.13 (0.76,1.68)
-	-	-	-	**PT**	0.96 (0.68,1.35)	1.21 (0.92,1.58)	1.12 (0.82,1.53)	1.18 (0.77,1.82)	1.21 (0.76,1.92)	1.22 (0.83,1.80)
-	-	-	-	-	**T-DM1P**	**1.26 (1.02,1.55)**	1.17 (0.82,1.67)	1.24 (0.83,1.83)	1.26 (0.82,1.93)	1.27 (0.90,1.80)
-	-	-	-	-	-	**PTC**	0.93 (0.70,1.24)	0.98 (0.70,1.37)	1.00 (0.69,1.46)	1.01 (0.76,1.34)
-	-	-	-	-	-	-	**LTC**	1.05 (0.68,1.63)	1.07 (0.67,1.72)	1.09 (0.73,1.62)
-	-	-	-	-	-	-	-	**T-DM1PC**	1.02 (0.73,1.41)	1.03 (0.67,1.59)
						-	-	-	**PTC_T-DM1P**	1.01 (0.63,1.62)
-	-	-	-	-	-	-	-	-	-	**T-DM1**

Results of treatment comparisons are read from right to left. For example, the risk ratio (95% confidence intervals) for breast conservative surgery of TC vs C is 0.99 (0.77,1.26). C = Chemotherapy; TC = Trastuzumab + chemotherapy, PC = Pertuzumab + chemotherapy; LC = Lapatinib + chemotherapy; PTC = Pertuzumab + trastuzumab + chemotherapy; LTC = Lapatinib + trastuzumab +chemotherapy; PT = Pertuzumab + trastuzumab; T-DM1P = trastuzumab emtansine + pertuzumab; T-DM1LC = trastuzumab emtansine +lapatinib + chemotherapy; T-DM1PC = trastuzumab emtansine + pertuzumab chemotherapy; PTC_T-DM1P = pertuzumab + trastuzumab + chemotherapy followed by trastuzumab emtansine + pertuzumab; T-DM1 = Trastuzumab emtansine.
